# Bio-Master: Design and Validation of a High-Throughput Biochemical Profiling Platform for Crop Canopies

**DOI:** 10.34133/plantphenomics.0121

**Published:** 2023-12-08

**Authors:** Ruowen Liu, Pengyan Li, Zejun Li, Zhenghui Liu, Yanfeng Ding, Wenjuan Li, Shouyang Liu

**Affiliations:** ^1^ Engineering Research Center of Plant Phenotyping, Ministry of Education, Jiangsu Collaborative Innovation Center for Modern Crop Production, Academy for Advanced Interdisciplinary Studies, Nanjing Agricultural University, Nanjing, China.; ^2^ College of Agriculture, Nanjing Agricultural University, Nanjing, China.; ^3^ State Key Laboratory of Efficient Utilization of Arid and Semi-arid Arable Land in Northern China, the Institute of Agricultural Resources and Regional Planning, Chinese Academy of Agricultural Sciences, Beijing, China.

## Abstract

Accurate assessment of crop biochemical profiles plays a crucial role in diagnosing their physiological status. The conventional destructive methods, although reliable, demand extensive laboratory work for measuring various traits. On the other hand, nondestructive techniques, while efficient and adaptable, often suffer from reduced precision due to the intricate interplay of the field environment and canopy structure. Striking a delicate balance between efficiency and accuracy, we have developed the Bio-Master phenotyping system. This system is capable of simultaneously measuring four vital biochemical components of the canopy profile: dry matter, water, chlorophyll, and nitrogen content. Bio-Master initiates the process by addressing structural influences, through segmenting the fresh plant and then further chopping the segment into uniform small pieces. Subsequently, the system quantifies hyperspectral reflectance and fresh weight over the sample within a controlled dark chamber, utilizing an independent light source. The final step involves employing an embedded estimation model to provide synchronous estimates for the four biochemical components of the measured sample. In this study, we established a comprehensive training dataset encompassing a wide range of rice varieties, nitrogen levels, and growth stages. Gaussian process regression model was used to estimate biochemical contents utilizing reflectance data obtained by Bio-Master. Leave-one-out validation revealed the model’s capacity to accurately estimate these contents at both leaf and plant scales. With Bio-Master, measuring a single rice plant takes approximately only 5 min, yielding around 10 values for each of the four biochemical components across the vertical profile. Furthermore, the Bio-Master system allows for immediate measurements near the field, mitigating potential alterations in plant status during transportation and processing. As a result, our measurements are more likely to faithfully represent in situ values. To summarize, the Bio-Master phenotyping system offers an efficient tool for comprehensive crop biochemical profiling. It harnesses the benefits of remote sensing techniques, providing significantly greater efficiency than conventional destructive methods while maintaining superior accuracy when compared to nondestructive approaches.

## Introduction

Plant biochemical components comprise elements such as water, nitrogen, chlorophyll, and dry matter [1]. Due to the complex interactions between the environment and canopy structure, there is noticeable heterogeneity in the vertical distribution of these biochemical components within the canopy. This variability results in functional differences among organs throughout the canopy’s vertical profile [2–5]. Thus, accurately monitoring the biochemical profile is essential for assessing crop functional status and understanding the coordination of physiological processes [6–8].

Traditional methods for estimating biochemical content typically involve destructive sampling of field leaves, followed by laboratory-based analysis [9]. For instance, dry matter and water content are deduced from the weight difference between fresh and dried samples [10]. Chlorophyll content is commonly determined via chemical extractions and spectrophotometry [11], while the Dumas method is frequently used for nitrogen content measurement [12]. These techniques are selected for their precision and reliability. Yet, they are labor-intensive, require specialized expertise, and have limited throughput [10,13,14]. The need to transport samples from field to laboratory before analysis further complicates the measurement process. This transition can significantly alter the biochemical content of samples, particularly water content. As a result, measurements from these traditional methods might not accurately represent the actual conditions of crops in the field.

Remote sensing technology offers a nondestructive alternative for estimating plant biochemical contents by leveraging hyperspectral reflectance, as the optical properties of leaves are largely influenced by their biochemical composition [7,15–21]. Building on this principle, various chlorophyll meters have been developed to efficiently determine leaf chlorophyll concentrations [22,23]. However, given the variability of biochemical contents among plants and within plant layers, measurements of leaf reflectance must be taken from multiple layers to yield a comprehensive biochemical profile. Consequently, the efficiency of this approach remains limited, hindering its operational use for assessing crop biochemical profiles.

In contrast to leaf reflectance, reflectance at the canopy scale, whether hyperspectral or multispectral, provides optical information over a more extensive area, making it more efficient for biochemical profiling of crop canopies. Yet, canopy reflectance emerges from a multifaceted interplay between the leaf’s biochemical contents, the three-dimensional (3D) structure of the canopy, and the soil background. This intricate relationship complicates the direct extraction of a biochemical profile from canopy reflectance data [24,25]. Especially, as crops mature and grow taller, incoming radiation struggles to penetrate the dense foliage. This approach tends to be limited to assessing biochemical content in the upper layers of the canopy [26].

While remote sensing-based nondestructive techniques currently outperform traditional destructive methods in efficiency, they are not capable of reliably estimating the entire canopy’s biochemical profile. This inadequacy necessitates destructive sampling to comprehensively profile the biochemicals [25]. Nevertheless, integrating automation can streamline this process, reducing labor and increasing efficiency. When this automation is synergized with remote sensing, efficiency is further improved. Accordingly, this research pursued three primary objectives: (a) to introduce Bio-Master, a high-throughput phenotyping system designed to automate sample processing and measurement; (b) to devise a machine learning model to estimate biochemical content, demonstrated using rice; and (c) to evaluate Bio-Master by monitoring the dynamic biochemical profiles in rice.

## Design of the Phenotyping System, Bio-Master

**Fig. 1.**
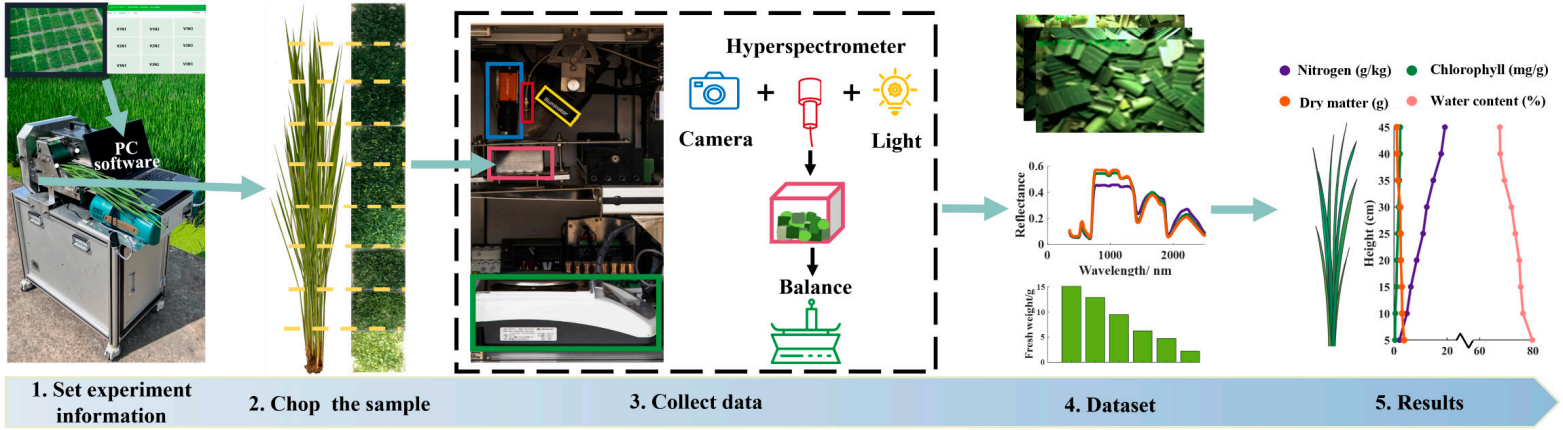
The flow chart of Bio-Master monitoring. The monitoring of Bio-Master is divided into five steps: first, set the experiment information on the software; second, quantitative chopping of the sample; third, collect sample data in the measurement chamber using sensors; fourth, view dataset; fifth, obtain the biochemical content results based on algorithms.

In this study, we developed Bio-Master, a phenotyping system designed to assess the biochemical components of plants on a segment-by-segment basis (Fig. 1). The system operates in three primary steps: It first segments the fresh plant and then further chops the segment into uniform small pieces. Then, it captures the hyperspectral reflectance of the sample within a dedicated measurement chamber. Finally, it offers segment-wise estimation of biochemical contents. This fully automated system, complemented by its user-friendly control software, stands out for its portability and ease of use, allowing for near-field measurements. It has dimensions of 680 mm × 400 mm × 960 mm and weighs 30 kg (Fig. 1).

### Hardware

The Bio-Master hardware is primarily composed of two main sections: the upper chopping area and the lower measurement chamber (Fig. 1). The design of the chopping mechanism drew inspiration from typical food processors used for vegetable chopping. Fresh plants collected from the field are transported by a conveyor belt into sharp blades that rotate at high speeds, slicing the plant material into small segments. The control software enables the adjustment of the chopped segment length, defaulting to 5 cm. The size of the chopped pieces can be modified by adjusting the blade rotation speed. The default rotational speed is set at 65 r/s, producing pieces approximately 8 mm in length.

Upon chopping, plant fragments settle into the designated sample container. These fragments are then positioned beneath the integrated light source and sensors within the measurement chamber (Fig. 1). Inside, a halogen lamp (70 W, 15 V, color temperature of 3,100 K, beam angle of 12°) ensures uniform and stable illumination. Accompanying this, a spectrometer (ASD Field Spec 4) with a wavelength accuracy of 0.5 nm and a fiber field of view of 25° captures the sample’s reflectance across the spectral range of 350 to 2,500 nm. Additionally, an RGB camera, with a resolution of 4,096 × 2,160, constantly monitors the sample’s condition. Once the reflectance data and images are acquired, the plant fragments are deposited onto an electronic balance (with a precision of 0.01 g) to determine their fresh weight. On average, assessing a 5-cm segment takes approximately 30 s under standard settings. It is important to highlight that samples, sourced directly from fields, might introduce mud into the sample container. To counter potential interference in reflectance measurements, the sample container’s bottom and sides are coated in black.

### Software

The software’s primary role is to govern the measurement procedure and document the collected data. For brevity, we will focus on its core functionality, and further details can be found in the Supplementary Materials (Figs. S1 and S2). Before initiating a measurement, users must either reopen or create a software project to input relevant experimental data, such as variety name, fertilizer application, growth phase, and plot design. All captured data, aligned with its project specifics, can be exported. Calibration for reflectance measurement is suggested after each machine restart. This can be achieved by positioning a white reference panel in the measurement chamber, manageable via the software.

### Preliminary test of the sample residue

**Fig. 2.**
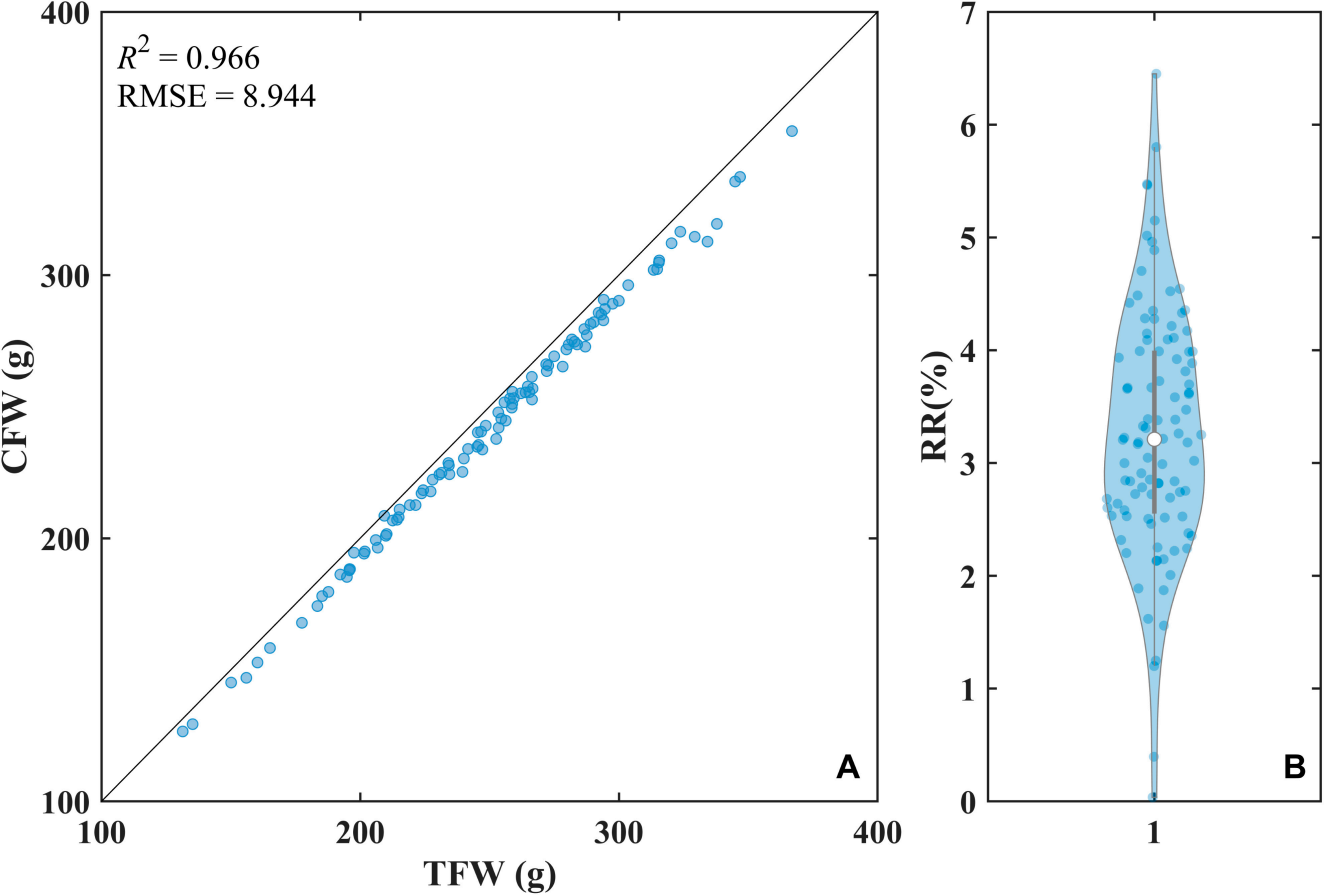
Assessment of the sample residue in Bio-Master. (A) Total fresh weight of individual plants (TFW) versus cumulative fresh weight measured by Bio-Master (CFW). (B) Boxplot of the relative residue.

Given that Bio-Master processes fresh field-collected plants, there is potential for moisture in the chopped samples, possibly leading them to adhere to the container. Our preliminary test aimed to determine the effect of such sample residues on weight accuracy. The test involved 100 rice plants, equating to over 1,000 segments. Individual plant’s total fresh weight (TFW) was assessed prior to processing. Bio-Master then documented the weight of each segment. All segment weights were aggregated to compute the cumulative fresh weight (CFW). Residual rates (RR) were then determined using Eq. 1. Results demonstrated strong consistency between TFW and CFW [*R*^2^ = 0.966, root mean square error (RMSE) = 8.944 g/sample; Fig. 2A], with over 75% of samples having an RR below 4% (Fig. 2B). This indicates minimal impact from sample residue, validating the weight measurement’s precision and laying a solid foundation for biochemical content estimation.

$$\mathrm{RR}=1-\left(\frac{\mathrm{CFW}}{\mathrm{TFW}}\right)\times 100\%$$ (1)

## Materials and Methods

### Field experiments

In 2021, two distinct field experiments were carried out in Danyang (26°22′N, 119°30′E), China. The first experiment involved three nitrogen levels (270, 135, and 0 kg hm^−2^) across three typical rice varieties, resulting in nine plots. Each plot spanned 2 m in width and 3 m in length. The second experiment planted 25 representative rice cultivars in plots measuring 1.5 m by 2 m, all receiving the same nitrogen treatment (270 kg hm^−2^). In total, the experiments encompassed 34 treatments, examining the effects of nitrogen levels, rice cultivars, and their combined influence on the biochemical profile.

Throughout the experiments, Bio-Master was employed for measurements at three critical growth stages: heading, flowering, and grain-filling. Representative rice samples, with average plant height and average number of stems from the plot, were gathered from the field and then measured using Bio-Master. Rice spikes were excluded after sampling due to their distinct structure and biochemical makeup, differentiating them from other plant parts. The study’s primary focus lay in determining the biochemical content profile of the rice’s vegetative organs. Overall, 100 plant samples were gathered across the three stages, totaling 1,806 segments.

### Measurement of the biochemical contents

To establish the training dataset, samples processed by Bio-Master were subsequently measured using traditional methods. Specifically, water content (C_w_, %) was calculated as the ratio of water to the TFW. The weight of dry matter (M_dry_, g) was determined via oven drying: first at 105°C for 30 min, then at 85°C until a constant weight was achieved. Chlorophyll content (C_ch_, mg/g) was gauged using the colorimetric method [27]. Nitrogen content (C_N_, g/kg) was assessed using the Kjeldahl method [28]. By aggregating the biochemical content of all segments from individual segments, contents at the plant scale, dry weight (CM_dry_, g), water content (CC_w_, %), nitrogen content (CC_N_, g/kg), and chlorophyll (CC_ch_, mg/g) were derived. Across the 34 treatments, both M_dry_ and C_w_ were gauged for all samples (1,806 in total). Additionally, 250 samples were chosen at random for C_N_ measurement, and 200 distinct samples were randomly selected to measure C_ch_.

### Model development and validation

In our study concerning estimation models, we explored two methodologies: a vegetation index (VI)-based model and a reflectance-driven model. Our results indicated superior performance from the reflectance-driven model compared to its VI-based counterpart. To maintain clarity and brevity, here will focus predominantly on detailing the reflectance-driven model (for specifics on the VI-based model, refer to Supplementary Materials 1.2). We gauged the biochemical content and assessed the estimation’s precision at the segment level. Within this context, we conducted a more in-depth evaluation of the estimation accuracy for dry matter and water content at the plant scale.

#### Estimation at segment scale

In this study, we employed the Gaussian Process Regression (GPR) approach as part of the reflectance-driven methodology. GPR is a nonparametric method that utilizes Gaussian process priors for regression analysis. Rather than relying on intermediate models or parameters, GPR directly constructs a flexible Bayesian model within the function space [29,30]. The measured reflectance, spanning 2,151 bands from 350 nm to 2,500 nm, served as the input, while the estimated biochemical content (M_dry_, C_w_, C_ch_, and C_N_) served as the output. Consequently, four separate GPR models were developed, each targeting a distinct biochemical content: M_dry_, C_w_, C_ch_, and C_N._

We compared three methods for the estimation of M_dry_, Direct, In-Direct, and Ensemble, respectively. In the Direct method, M_dry_ estimation model was built using GPR model with the input of reflectance (M_dry_dir_, g). Then, for the In-Direct method, we estimated C_w_ (C_w_^est^, %) first and then used the measured fresh weight (FW, g) recorded by Bio-Master to estimate M_dry_ (M_dry_in_, g) (Eq. 2). Finally, the ensemble approach would merge the estimates from the Direct and In-Direct method (M_dry_en_, g). Precisely, the contribution of M_dry_in_ and M_dry_dir_ to M_dry_in_ was weight by the relative value of their RMSE (RMSE_dir_: RMSE of the Direct method, RMSE_in_: RMSE of the In-Direct method) (Eq. 3).

$$M_{\mathrm{dry}\_\text{in}}=\left(1-{C_{w}}^{\mathrm{est}}\right)\times \mathrm{FW}$$ (2)

$$\begin{array}{c} M_{\mathrm{dry}\_\mathrm{en}}=\frac{{\text{RMSE}}_{\mathrm{dir}}}{{\text{RMSE}}_{\mathrm{dir}}+{\text{RMSE}}_{\text{in}}}\times M_{\mathrm{dry}\_\text{in}}\\ \;+\frac{{\text{RMSE}}_{\text{in}}}{{\text{RMSE}}_{\mathrm{dir}}+{\text{RMSE}}_{\text{in}}}\times M_{\mathrm{dry}\_\mathrm{dir}} \end{array}$$ (3)

#### Estimation at plant scale

For both M_dry_ and C_w_, we determined the actual values for all samples. However, for the other two components, only a subset of samples was randomly selected for measurement. This approach facilitated the performance evaluation of M_dry_ and C_w_ estimations at the plant scale, denoted as CM_dry_ and CC_w_, respectively. Specifically, the measured CM_dry_ represented the dry weight of the entire plant, while the estimated CM_dry_ was computed by summing the estimated M_dry_ of each segment for the entire plant. Consequently, we derived three distinct CM_dry_ estimations based on the three aforementioned M_dry_ estimation methods. As for CC_w_, its actual value was derived using the measured fresh weight of individual plants and the corresponding measured CM_dry_. The estimated CC_w_ (CC_w_^est^, %) was determined by dividing the total segment water weight by the total segment fresh weight for each plant. The fresh weight and corresponding water weight for each segment were deduced from the estimated M_dry_ and C_w_ values.

#### Model validation

The model’s performance was validated using the leave-one-out cross-validation method [31,32]. For each of the four biochemical components, we trained the GPR model 34 times, each time using data from 33 treatments and leaving out one treatment for validation. Model efficacy was assessed using the coefficient of determination (*R*^2^) and the RMSE between the actual and predicted biochemical content. Validation was conducted at the segment scale for all four components and extended to the plant scale for both CM_dry_ and CC_w_.

### Analysis of the biochemical profile

After validating the estimation models, we estimated the biochemical content for all samples to analyze the biochemical profiles. Given the varying rice plant heights across treatments, the number of measured segments differed. To standardize comparisons among treatments, we converted absolute height to relative height, with 0 representing the plant base and 1 the top. All treatments were then interpolated into 10 uniformly spaced segments. Subsequently, we calculated the average biochemical content and standard deviation across the vertical profile. We also analyzed correlations among the four biochemical components and investigated the impact of cultivar, nitrogen treatments, and growth stages on these correlations using analysis of variance. All analyses were performed using MATLAB R2021a.

## Results

### Accuracy of the model estimation

**Fig. 3.**
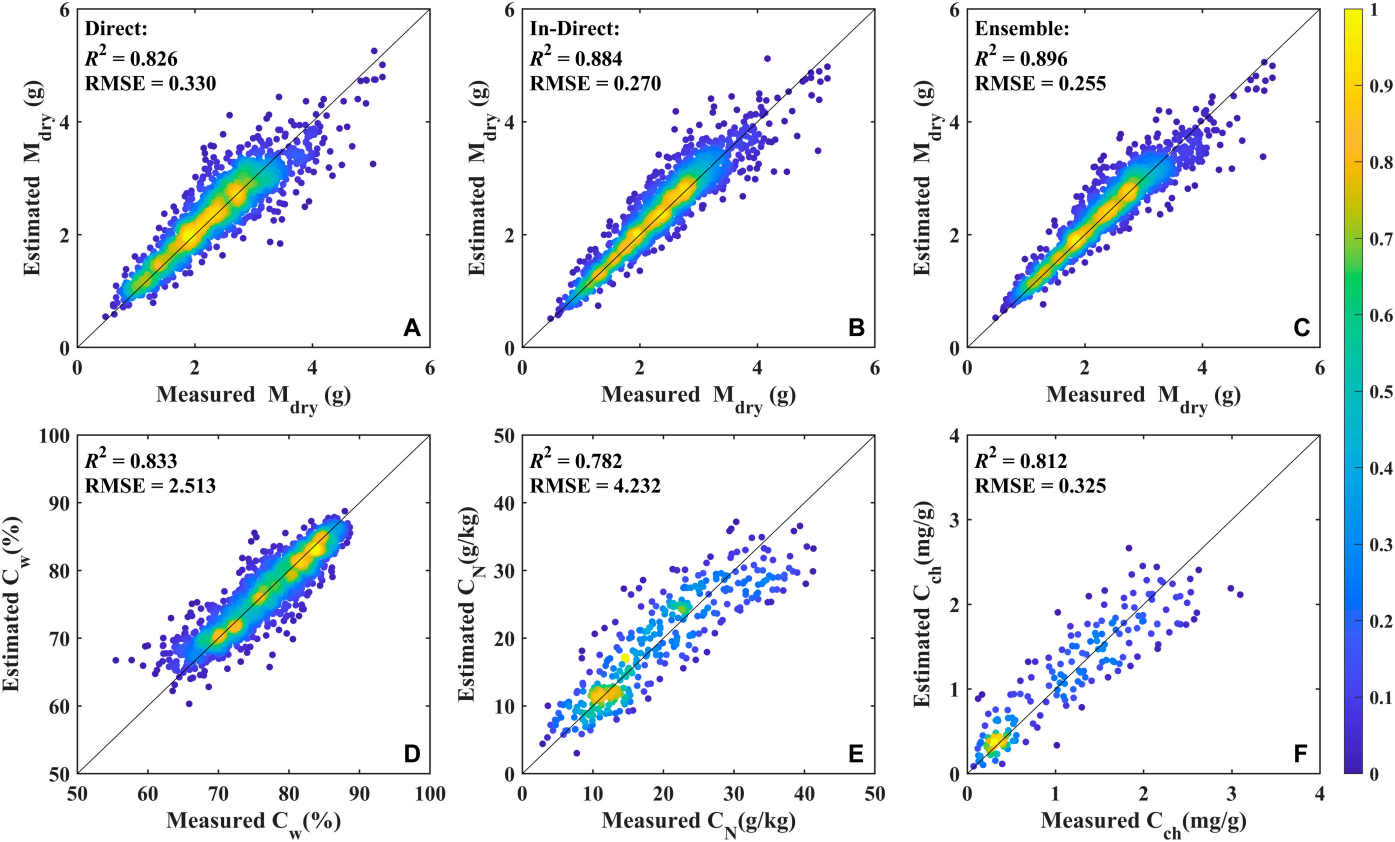
Validation of the estimation performance at segment scale. M_dry_ (dry weight, g) validation results using the Direct (A), In-Direct (B), and Ensemble (C) methods with sample size 1,806. (D) C_w_ (water content, %) validation results with sample size 1,806. (E) C_N_ validation results with sample size 250. (F) C_ch_ validation results with sample size 200. The color bar indicates the density of samples.

The M_dry_, C_w_, C_N_, and C_ch_ estimates using the GPR model at the segment scale are shown in Fig. 3. There was a high consistency in M_dry_ estimated using the three different methods (Direct: *R*^2^ = 0.826, RMSE = 0.330 g; In-Direct: *R*^2^ = 0.884, RMSE = 0.270 g; Ensemble: *R*^2^ = 0.896, RMSE = 0.255 g). However, the Ensemble method had the most consistent performance with minimal variation in M_dry_. For the other three components, quite satisfactory estimation accuracy was also achieved (C_w_: *R*^2^ = 0.833, RMSE = 2.513 %; C_N_: *R*^2^ = 0.782, RMSE = 4.232 g/kg; C_ch_: *R*^2^ = 0.812, RMSE = 0.325 mg/g). The estimation accuracy was slightly degraded when the M_dry_ values were high, or C_w_ values were low, which could be due to the unbalanced distribution of the training dataset. By contrast, the estimation accuracy of C_N_ and C_ch_ was lower than that of M_dry_ and C_w_, possibly because the size of the training dataset for C_N_ and C_ch_ was much smaller than that of M_dry_ and C_w_.

**Fig. 4.**
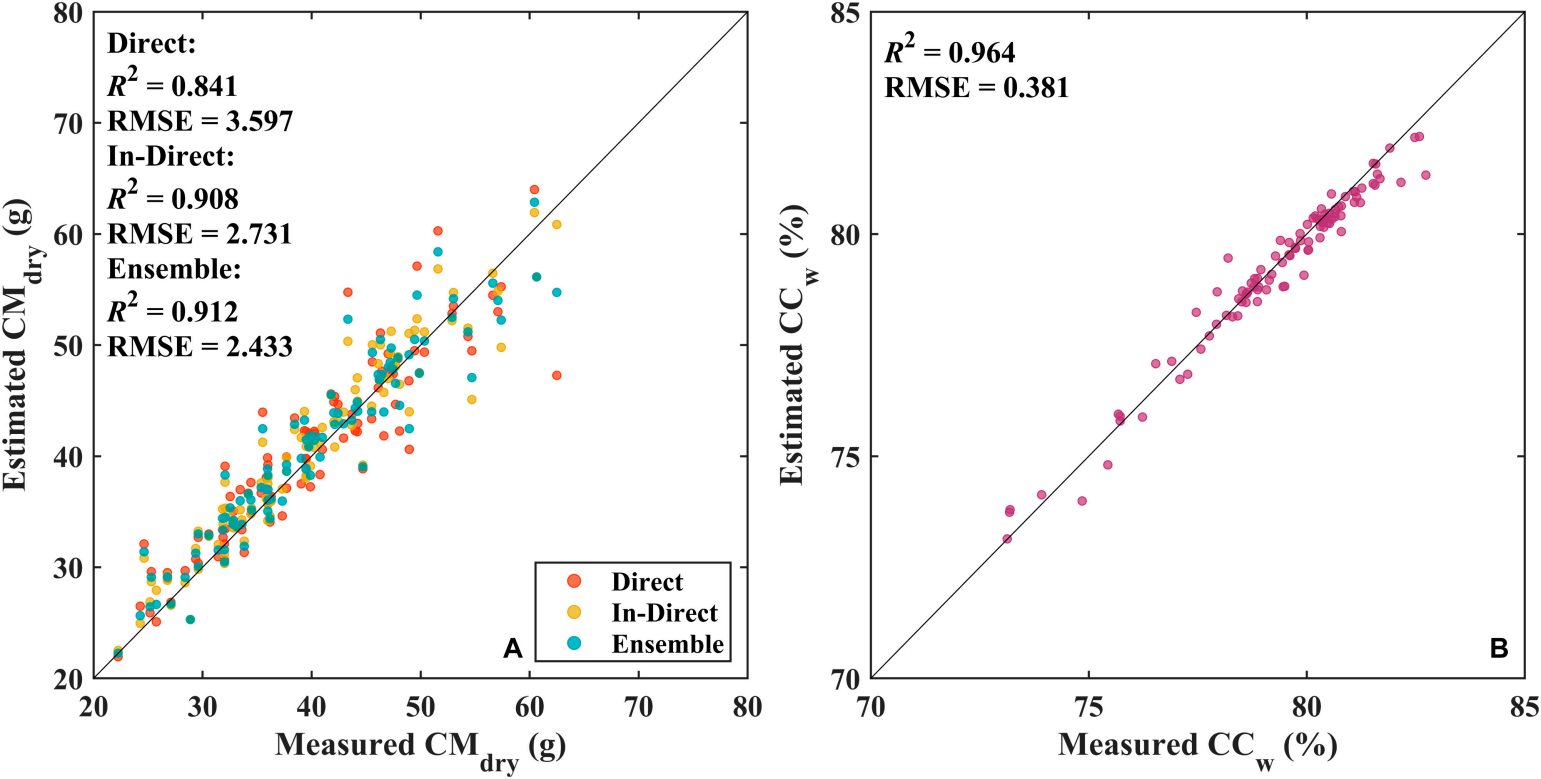
Validation of the estimation performance at the plant scale. (A) Validation of CM_dry_ estimated using the Direct, In-Direct, and Ensemble methods with sample size 100. (B) Validation of the estimated CC_w_ with sample size 100.

At the plant scale, CM_dry_ and CC_w_ estimated using the GPR model are shown in Fig. 4*.* For CM_dry_, the three methods we tested performed well (Direct: *R*^2^ = 0.841, RMSE = 3.597 g; In-Direct: *R*^2^ = 0.908, RMSE = 2.731 g; Ensemble: *R*^2^ = 0.912, RMSE = 2.433 g). Besides, these accuracy results were consistent with those at the segment scale, with the Ensemble method recording the most consistent performance with minimal variation in CM_dry_. However, in the high-value interval, the estimated CM_dry_ was generally underestimated (Fig. 4A) due to the large sample size in the low-value interval and the small sample size in the high-value interval. At the same time, the accuracy for the CC_w_ estimation model was very high (*R*^2^ = 0.964, RMSE = 0.381 %; Fig. 4B). These results imply that our method efficiently estimates the dry weight and water content for rice crops at the plant scale.

### Vertical profile of biochemical contents

**Fig. 5.**
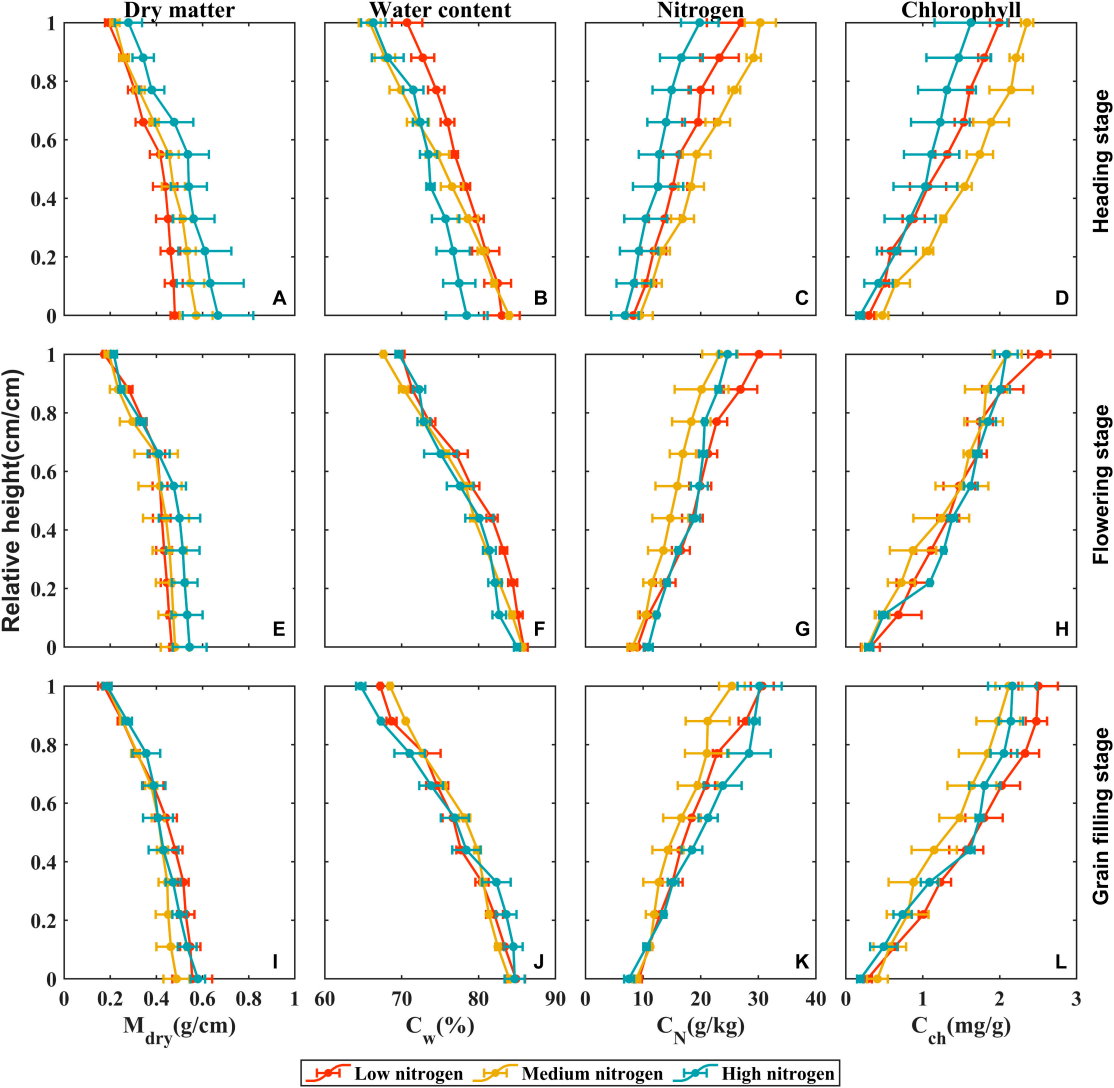
Profile distribution of biochemical contents in rice at different stages. Vertical profile of mean (A) C_w_ (water content, %) (B) M_dry_ (dry weight, g) (C) C_N_ (nitrogen content, g/kg), and (D) C_ch_ (chlorophyll content, mg/g) along the relative plant height under different nitrogen treatments at the heading stage, then for the flowering stage (E to H), and for the grain-filling stage (I to L). Error bars show the standard deviation of the three replicates, corresponding to three varieties.

An assessment of the vertical distribution pattern of biochemical contents in rice is shown in Fig. 5. M_dry_ and C_w_ were consistently increased from the top to the bottom of rice plants, while C_N_ and C_ch_ were decreased in all three nitrogen treatments and phenological stages. Specifically, the C_w_, C_N_, and C_ch_ profiles were roughly linear, whereas the M_dry_ profile change was linear in the top parts but relatively constant in the lower parts. At the heading stage, M_dry_ was increased from the low to the high nitrogen treatments (Fig. 5A), which contrasted with the C_w_ (Fig. 5B). However, there were inconsistencies in C_N_ and C_ch_ at a given plant height among the different nitrogen treatments. This implies that the impact of nitrogen on the biochemical profile is more directly reflected by M_dry_ and C_w_. Besides, the difference in biochemical profiles was more distinct among the nitrogen treatments at the heading stage than later stages. In particular, the M_dry_ and C_w_ profiles almost converge with that at the grain-filling stage (Fig. 5I and J).

### Correlation among the biochemical components at segment scale

**Fig. 6.**
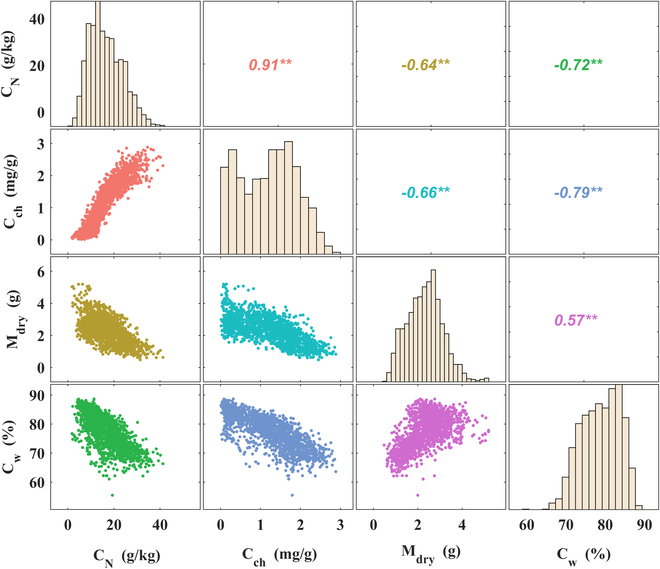
Correlation among the biochemical contents at segment scale. ** indicates an extremely significant correlation (*P* < 0.01). − and + indicate a negative correlation and a positive correlation, respectively. The analysis was conducted over the entire dataset considering the variation of cultivar, nitrogen treatment, and phenology with sample size 1,806. C_w_ (water content, %), M_dry_ (dry weight, g), C_N_ (nitrogen content, g/kg), and C_ch_ (chlorophyll content, mg/g).

Figure 6 shows the correlation among the four biochemical components at the segment scale across the entire dataset with 34 treatments. We found that the contents of all these components were significantly correlated. Therein, C_N_ and C_ch_ were significantly positively correlated with a correlation coefficient of 0.91. Besides, C_N_ and C_ch_ negatively correlated with both M_dry_ and C_w_ and the correlation with C_w_ was relatively stronger. In addition, M_dry_ and C_w_ were positively correlated with a correlation coefficient of 0.57.

**Fig. 7.**
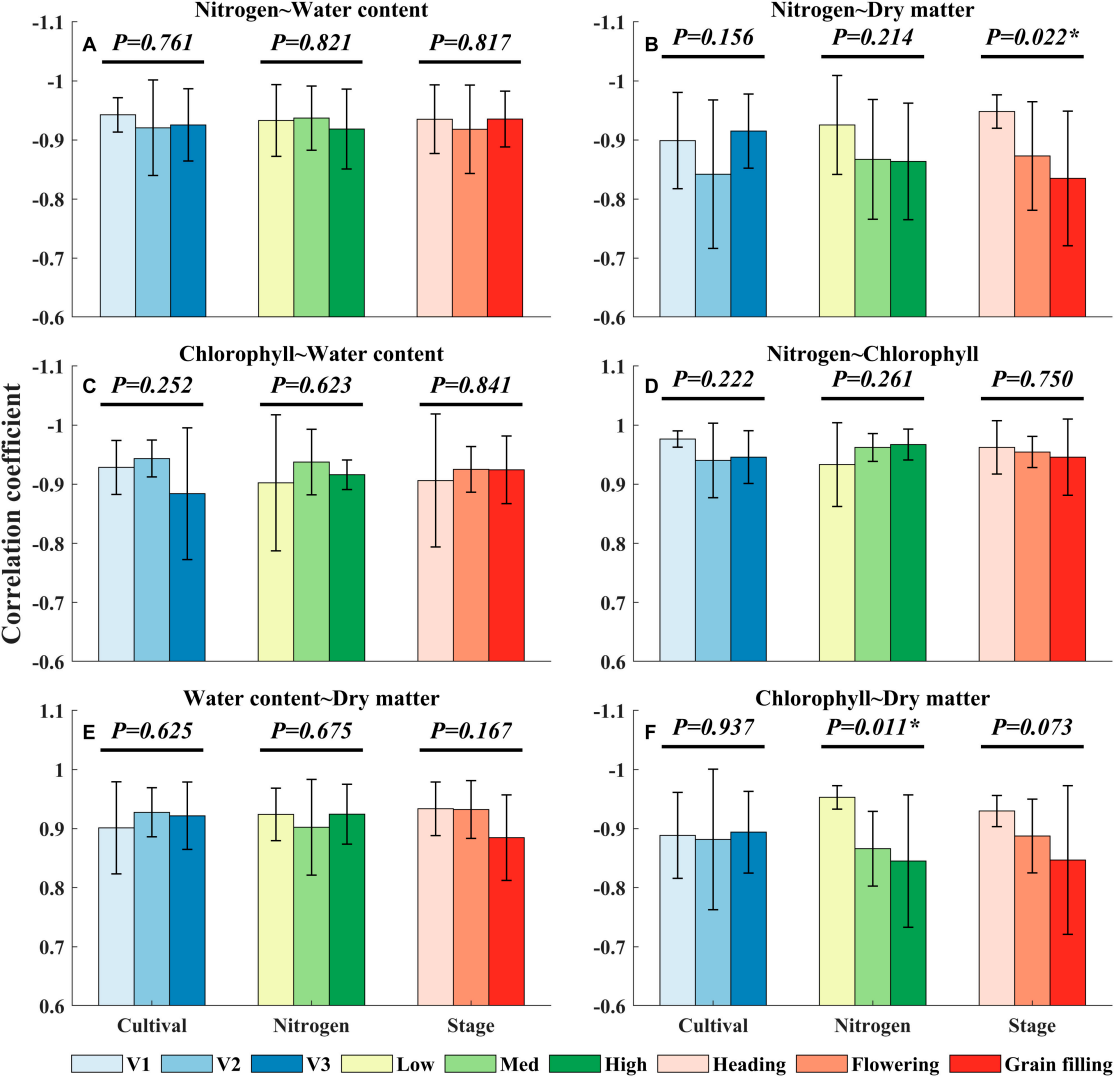
Effects of cultivar, nitrogen treatment, and phenology on the correlation among the biochemical contents. The experiment included three cultivars by three nitrogen levels across three stages. * indicates that the factor impacts the correlation significantly (0.01 < *P* < 0.05).

We further inspected the impact of variety, nitrogen treatment, and phenology on the correlation among the biochemical contents. Figure 7 shows that most of the correlation was not significantly affected by the factors considered. Exceptionally, the correlation between C_N_ and M_dry_ was significantly decreased from the heading to the grain-filling stages (Fig. 7B, *P* < 0.05). A similar trend was observed in the correlation between C_ch_ and M_dry_, although it was not significant (Fig. 7F, *P* > 0.05). Moreover, the correlation between M_dry_ and C_ch_ was significantly decreased with the increment of nitrogen treatment (Fig. 7F, *P* < 0.05). Similarly, the correlation coefficient between C_w_ and M_dry_ gradually decreased across the growth period (Fig. 7E). In contrast, C_N_ and C_ch_ always had a very significantly high correlation with a slight increment in nitrogen treatment (Fig. 7D).

### Biochemical contents at the plant scale

**Fig. 8.**
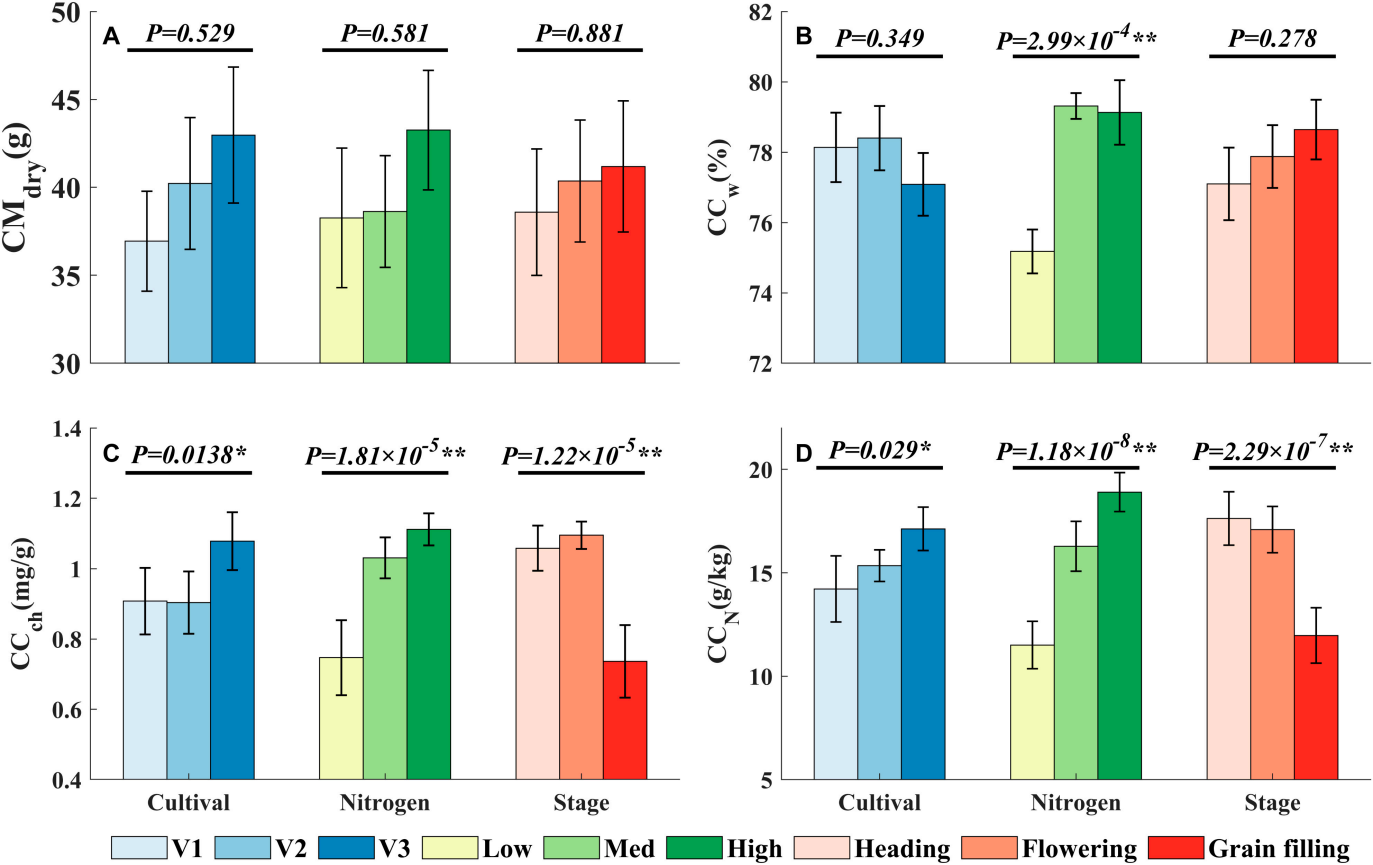
Effects of different cultivars, nitrogen treatments, and growth stages on CM_dry_ (A), CC_w_ (B), CC_ch_ (C), and CC_N_ (D). The bars represent the mean CC_w_, CM_dry_, CC_N_, and CC_ch_. The error bars represent the standard deviation. The *P* value represents the probability that a significant effect of the factor (cultivar, nitrogen level, and stage) is observed. *P* < 0.01 indicates an extremely significant impact (**). 0.01 < *P* < 0.05 indicates a significant impact (*).

In most cases, the cultivar, nitrogen treatment, and phenological stage had a significant effect on the biochemical contents at the plant scale (Fig. 8). In particular, CC_ch_ (Fig. 8C) and CC_N_ (Fig. 8D) were significantly different among the three cultivars (0.01 < *P* < 0.05), increased significantly with increasing nitrogen application (*P* < 0.01), and decreased significantly with advancing phenological stage (*P* < 0.01). CC_w_ (Fig. 8B) varied significantly among the three nitrogen treatments, but it did not reach a significant level between varieties and phenological stages. Exceptionally, CM_dry_ (Fig. 8A) was relatively little affected by the three factors mentioned above. Although it gradually increased with increasing nitrogen application, and advancing phenological stage, none reached a significant level (*P* > 0.05). In summary, CM_dry_ and CC_w_ were less affected by variety, nitrogen treatment, and phenological stage, while CC_ch_ and CC_N_ were more affected by the above factors, especially nitrogen treatment and phenological stage, and the changes of the two components were consistent under the influence of these factors.

## Discussion

### Advantages of Bio-Master

Following comprehensive testing across a range of conditions, Bio-Master, developed in this study, has proven itself capable of accurately estimating water, dry matter, chlorophyll, and nitrogen contents in rice, at both the segment and plant scales. This system provides the flexibility to customize the length of segments to be measured, allowing for precise control over the level of detail in canopy profiles. One key advantage of Bio-Master lies in its portability and field-friendliness. It can be effortlessly transported to near the field, where measurements can be obtained after plant sampling. This minimizes the risk of alterations in biochemical components that can occur during the transportation of samples from the field to the laboratory and subsequent pre-processing. Moreover, Bio-Master efficiently processes fresh samples and concurrently estimates the four biochemical contents within just 30 s per segment. By contrast, the conventional methods may take hours for specific sample pre-processing. In this aspect, Bio-Master enhances efficiency by a factor of a hundred.

Note that some may critique this methodology as it still necessitates plant collection from the field, making it a destructive method. In contrast, remote sensing techniques using multi-spectral images allow for the nondestructive derivation of biochemical contents from canopy reflectance [33]. Undoubtedly, these nondestructive methods excel in terms of efficiency. However, they are often susceptible to the influences of soil backgrounds, lighting conditions, and canopy structures. Additionally, the heterogeneity of biochemical components interacting with the canopy structure complicates canopy reflectance, affecting the accuracy of these methods in estimating biochemical contents. Conversely, Bio-Master is designed to first homogenize plant segments into consistent small pieces, thus harmonizing the canopy structure. Subsequently, it measures the reflectance of the processed sample in a controlled darkroom environment to ensure uniform lighting conditions. Overall, Bio-Master combines the advantages of remote sensing techniques to achieve significantly greater efficiency than conventional destructive methods, while maintaining superior accuracy compared to nondestructive methods.

### Estimation models

The estimation model was developed using GPR trained with the measured dataset of the four biochemical components. Alternatively, rather than using measurements, it is possible to train the model with simulations [15,34]. Theoretically, this strategy saves time in establishing the training dataset while potentially increasing the model’s robustness. However, specific to the estimation of biochemical contents, it has been demonstrated that the model trained with measurements has superior performance compared with the model trained with simulations [18,35]. This can primarily be attributed to that current simulation models are not realistic enough to represent the complicated 3D canopy architecture. Besides, the GPR algorithm was selected because Camacho et al. [35] verified that it performed better than other popular machine learning techniques for biochemical estimations, including the artificial neural networks and support vector machine regression. Even so, GPR for empirical training should be done with caution due to its potential degradation when applied to conditions dissimilar to the training dataset. This study collected the training dataset at the heading, flowering, and grain-filling stages. Therefore, it is important to include data from the other growth stages in the training dataset before applying the model to the other growth stages.

### Potential improvements

We tested the Bio-Master phenotyping system on rice crops as our initial proof of concept. To adapt its utility to a broader range of crops, we need to consider both the structural aspects of the measured plant and the development of crop-specific estimation models. First, the suitability of the plant’s size and canopy structure for processing into smaller pieces must be ensured. Our methodology can be readily applied to crops like wheat, barley, and others with similar plant structures as rice. However, for larger crops such as maize and sorghum, some modifications to the chopping mechanism may be required. Second, the creation of crop-specific estimation models is essential for extending this approach to other crops.

It is crucial to acknowledge that our current methodology can estimate the biochemical profiles of the entire canopy during the vegetative stage. Given the distinct biochemical profiles of various plant organs, there is a notable interest in separately measuring them. The structure of the panicle, for instance, remains significantly different even after chopping into pieces, and including it in a sample with other organs would introduce significant estimation uncertainties. To enhance estimation accuracy, our methodology is presently limited to growth stages preceding panicle appearance. However, the system is equipped with a high-resolution camera that opens the possibility of estimating the proportion of each organ based on images. Subsequently, we can develop an estimation model that combines the reflectance and composition of the sample, potentially enabling the separate estimation of biochemical contents for each plant organ.

Compared to conventional methods, our methodology enhances the efficiency of measuring the four biochemical components by a factor of a hundred. Nevertheless, further efforts are essential to streamline the measurement of hundreds of genotypes for applications in plant breeding and genome-wide association studies. First, there is room for improvement in efficiency by optimizing the mechanical components of Bio-Master. With these enhancements, we can aim to measure 100 genotypes within a matter of hours. Additionally, the high cost of the system poses a limitation to its widespread adoption. The major cost contributor is the high-grade spectrometer, featuring 2,151 bands spanning from 350 to 2,500 nm. However, it is worth noting that some of these bands are redundant when estimating the biochemical components. By identifying critical sensitive bands for each biochemical component, subsequently we can replace the costly spectrometer with a more budget-friendly alternative.

### The biochemical profiles

Our results showed that the vertical distribution of the four biochemical contents changed linearly, and M_dry_ and C_w_ gradually decreased from the bottom to the top of the plant, while the nitrogen content and chlorophyll content gradually increased. Variety, nitrogen treatment, and growth period did not change the trend of variation, but affected the absolute content of each level, which is consistent with previous findings using conventional methods [25,36–38]. This further validates the reliability of Bio-Master.

We observed that variations in nitrogen fertilizer application could influence the shifts in the biochemical content profile. This phenomenon can be attributed to the fact that, under low nitrogen application rates, the nutrients supplied by the upper leaves of the crop fail to meet the reservoir’s demands, resulting in the lower leaves redistributing nutrients to the upper portion of the crop. This, in turn, restricts canopy expansion. An appropriate increase in nitrogen fertilizer can enhance canopy structure and improve light penetration within the lower layer of the population [39]. Furthermore, our findings indicated that as the growth stage progresses, the response of each biochemical component to nitrogen gradually diminishes. The vertical distribution curve of biochemical components under various nitrogen application levels approaches a uniform level. This may be due to the gradual transfer of nutrients to the rice ear during the late growth stage. In the current study, a strong positive correlation was observed between C_w_ and M_dry_, and this relationship remained largely unaffected by factors such as cultivar, growth stage, and phenology. In contrast, the response of C_ch_ and C_N_ to nitrogen levels exhibited a more complex behavior. This suggests that biomass and water content might be the more sensitive indicators for diagnosing the crop’s nitrogen status.

## Conclusion

We developed the phenotyping system named as Bio-Master, designed for the comprehensive measurement of biochemical profiles in crops. This system is capable of directly processing fresh plant samples and simultaneously determining the content of four key biochemical components: water, dry matter, chlorophyll, and nitrogen. Our evaluation, particularly in rice crops, has demonstrated that Bio-Master can deliver accuracy comparable to the referenced destructive method while improving efficiency by a remarkable factor of a hundred. What sets Bio-Master apart is its capacity to perform measurements right in the field shortly after sample collection, effectively minimizing alterations in plant status during transportation and processing. Consequently, the estimates generated by Bio-Master are more likely to closely reflect in situ values. While our initial testing focused on rice, this methodology can be readily extended to other crops such as wheat and barley. This extension primarily necessitates the collection of a measurement dataset to construct the estimation model. In future research, we plan to explore the integration of information from RGB images into the estimation model, particularly for assessing the biochemical contents of plant organs. In summary, the Bio-Master phenotyping system, as developed in this study, represents an efficient and powerful tool for characterizing the biochemical profiles of crops. This advancement holds great potential for investigating the intricate interplay between genotype and environment in shaping the dynamics of biochemical distribution.

## Data Availability

The data supporting the findings of this study are available from the corresponding author upon reasonable request.
