# Supplementary material for: Bio-Master: Design and Validation of a High-Throughput Biochemical Profiling Platform for Crop Canopies
Source: Plant Phenomics. 2023 Dec 8;5:0121. doi: 10.34133/plantphenomics.0121 (PMC10709073; doi:10.34133/plantphenomics.0121)
Supplement: Supplementary 1 — Figs. S1 to S3 Table S3 [file plantphenomics.0121.f1.docx]

*Supplementary Materials*

**High-throughput biochemical profiling of crop canopies near the field**

Ruowen Liu, Pengyan Li, Zejun Li, Zhenghui Liu, Yanfeng Ding, Wenjuan Li, Shouyang Liu

## Operation instruction of the Bio-Master

The Bio-Mater software comprises three sequential steps, organized by temporal sequence and functional logic (Figure S1). The flowchart of the software interface is shown in the Figure S2. The preparation phase encompasses preparatory tasks, including connecting to the wireless network, logging into the software (Figure S2a), and selecting the experimental project (Figure S2b). Users can either reopen an existing project or create a new one, necessitating the input of location, variety, and growth stage (Figure S2c). Additionally, users can define a district layout by naming each district and inputting related information. During the experimental phase, users select the project (Figure S2d), set the segmenting length and chopping degree, and then start the measurement (Figure S2e). When the measurement is done, all the project-related data can be exported (Figure S2f). This facilitates subsequent data viewing, management and processing.


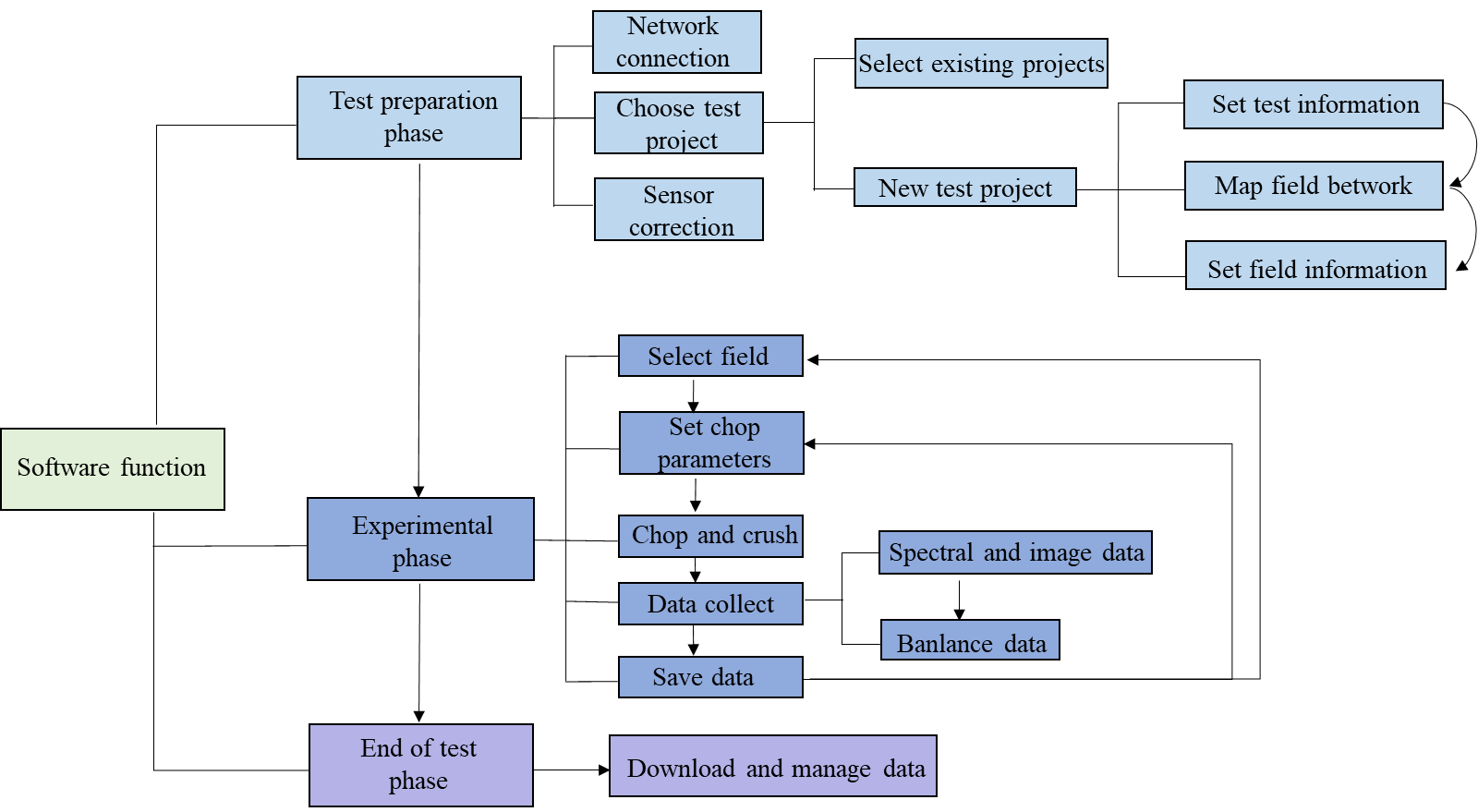


Figure S1. Function flow of the Bio-Master software


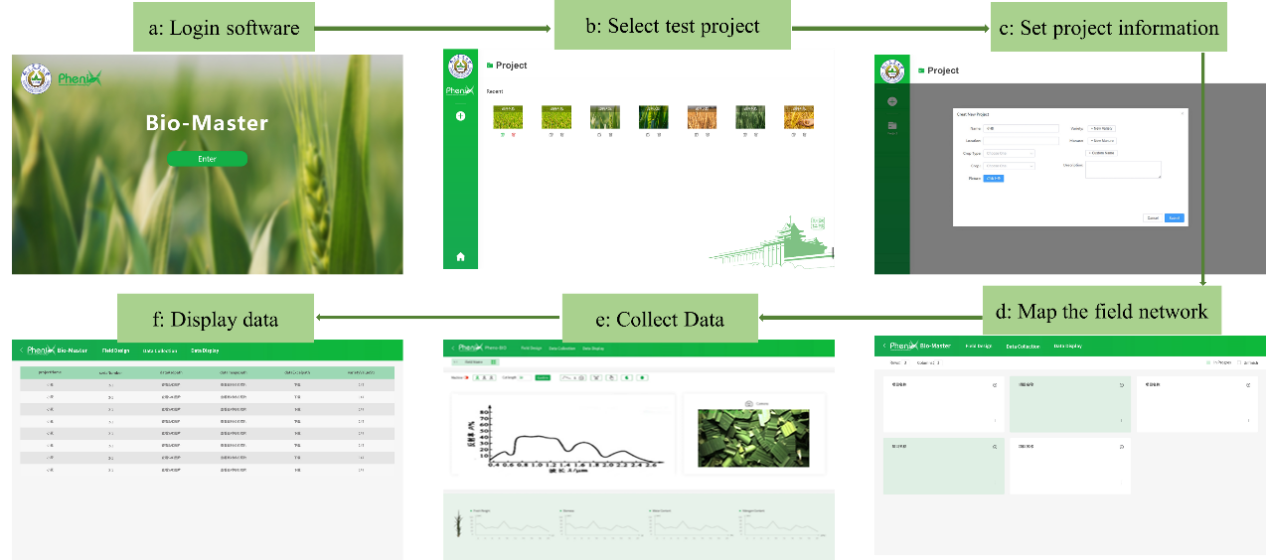


Figure S2. Illustration of the steps to conduct Bio-Master measurements with its software

## VI based biochemical estimation

In our research, we utilized the difference vegetation index (DVI), ratio vegetation index (RVI), and normalized vegetation index (NDVI) to develop a novel spectral index, derived from the original spectral reflectance spanning 350-2500 nm. Then a simple linear fitting model was used to estimate M_dry_, C_w_, C_N_ and C_ch_. The leave-one-out cross-validation approach was used to evaluate its estimation performance that alignes in the GPR-based model (described in the Section 2.3). The performance of the optimal band combination to estimate different biochemical components of rice are shown in Table S3 and Figure S3. Results indicate variability in the estimation capabilities of vegetation indices for different biochemical components, showcasing the highest precision for C_ch_ and the least for M_dry_. Nonetheless, the average accuracy lags behind that of the GPR-based model (shown in Figure 3).


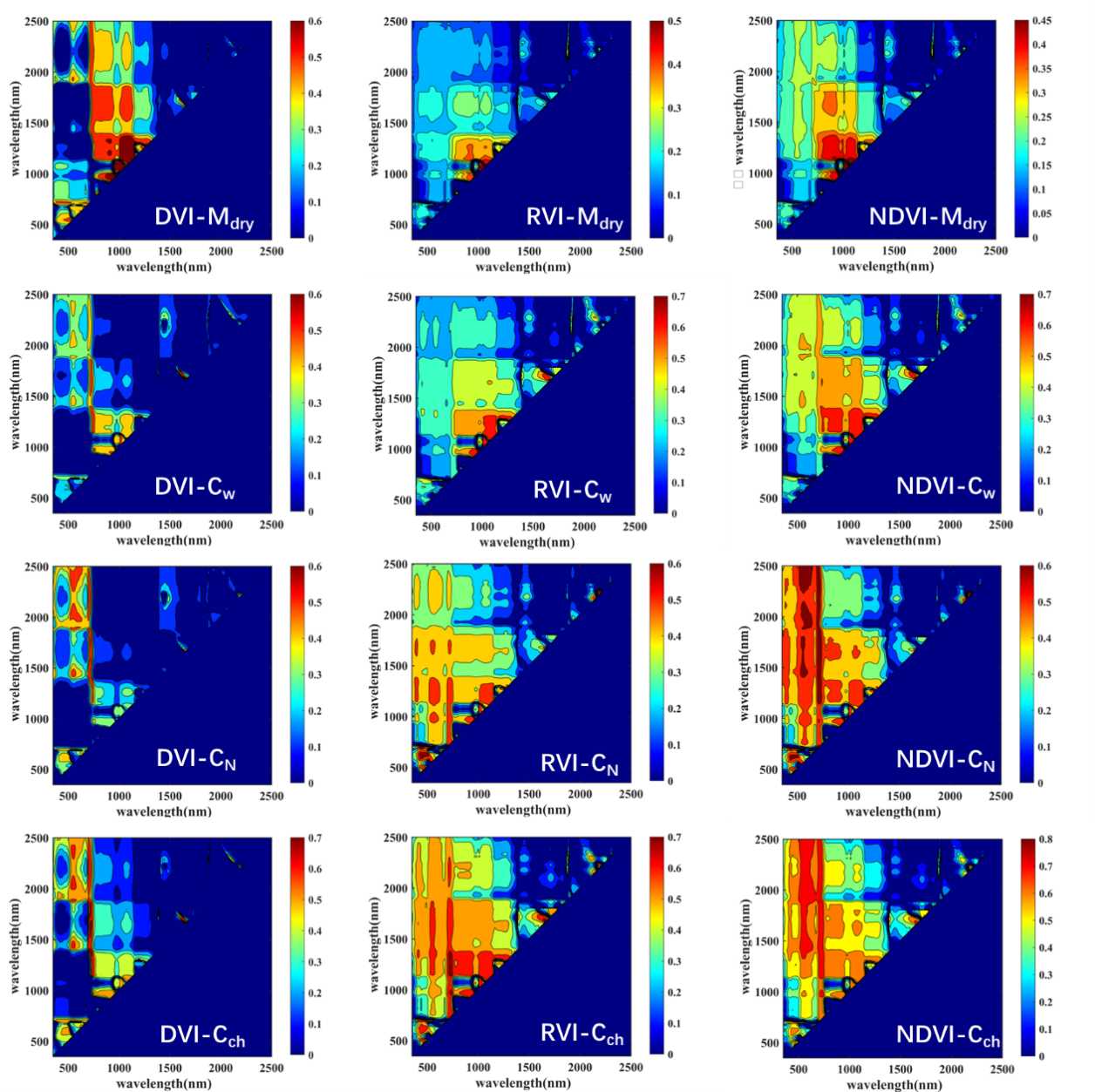


Figure S3: Performance of the biochemical estimates using various vegetation indices (VIs). The color indicates the coefficient of determination. Indices include: Difference vegetation index (DVI), ratio vegetation index (RVI), and normalized vegetation index (NDVI).

Table S3: Optimal band combination and model effect of self-constructed VI for estimating different biochemical components in rice.

| Biochemical components | Vegetation index | Model determination coefficient R2 | |
| --- | --- | --- | --- |
|  |  | \| Training \| Test \| \| --- \| --- \| | |
| M_dry_ | DVI_(1010, 1020)_ | 0.683 | 0.616 |
|  | RVI_(1030, 1070)_ | 0.503 | 0.485 |
|  | NDVI_(1030, 1070)_ | 0.499 | 0.480 |
| C_w_ | DVI_(1650, 1680)_ | 0.680 | 0.693 |
|  | RVI_(1660, 1680)_ | 0.703 | 0.705 |
|  | NDVI_(1660, 1680)_ | 0.730 | 0.704 |
| C_N_ | DVI_(720, 1770)_ | 0.604 | 0.541 |
|  | RVI_(450, 630)_ | 0.577 | 0.481 |
|  | NDVI_(710, 2320)_ | 0.671 | 0.597 |
| C_ch_ | DVI_(730, 1370)_ | 0.723 | 0.638 |
|  | RVI_(730, 1160)_ | 0.742 | 0.730 |
|  | NDVI_(710, 1890)_ | 0.804 | 0.786 |
